# Exnovation: A Concept Analysis

**DOI:** 10.1002/nop2.70349

**Published:** 2026-01-09

**Authors:** Abdulqadir J. Nashwan, George V. Joy, Kamaruddeen Mannethodi, Jibin Kunjavara, Fadwa Alhalaiqa, Albara Mohammad Ali Alomari, Ahmed A. Abujaber

**Affiliations:** ^1^ Nursing Department Hamad Medical Corporation Doha Qatar; ^2^ College of Nursing Qatar University Doha Qatar; ^3^ College of Health Science University of Doha Doha Qatar

**Keywords:** concept analysis, exnovation, health care innovation, innovation, nursing

## Abstract

**Aim:**

To conduct an in‐depth concept analysis of exnovation, exploring its significance, conceptual mechanisms and impacts in administration, business and healthcare, particularly emphasising its relevance to nursing.

**Background:**

Exnovation is applicable in diverse fields, including information technology, manufacturing, business, education and cultural contexts; however, its utilisation in nursing and healthcare is limited and not widely adopted.

**Data Sources:**

The literature for this concept analysis was retrieved from two databases, PubMed and Scopus. A systematic search approach was employed across studies from business, administration and health care, specifically without restriction based on the year of publication.

**Review Methods:**

The article utilised Walker and Avant's 8‐step approach to concept analysis, which entailed identifying the applications of the concept in various settings, its fundamental conceptual characteristics and developing model, borderline, related and contradictory instances. Additionally, the antecedents, consequences and empirical references of exnovation in nursing were established.

**Results:**

The analysis revealed both onomasiological approaches to elucidate the evolution of innovation terminology and a semasiological approach to explain the concept across various contexts. Moreover, it identified antecedents to innovation in nursing, such as technological advancements and the adoption of evidence‐based practices (EBPs), while also delineating consequences primarily focused on enhancing quality patient care and job satisfaction.

**Conclusion:**

As exnovation emerges as a novel concept in nursing and medical practice, further research is warranted to tackle the recognised limitations and formulate practical guidelines for effectively integrating exnovation within nursing and healthcare settings.

## 1 Introduction

Concept analysis involves a systematic and thorough approach to examining, clarifying, validating, defining and distinguishing an abstract concept from similar ideas to support theory development and interpretation of abstract concepts (Walker and Avant 2005). The concept finds application across various disciplines, such as psychology, sociology, nursing and education. It involves breaking down complicated ideas into basic parts to help with implications, applications and relationships within a particular context (Scott Tilley 2008). Concepts are sometimes labelled as ‘the cornerstones of theory’ (Walker and Avant 2005, 26), and some published concept analyses suggest that concepts can be productively developed before significant theorising. However, this viewpoint has been subject to critique within nursing and philosophy. Critics argue that concepts are more influenced by existing theory rather than being the driving force behind theory development (Risjord 2009).

The primary objective of conducting a concept analysis on exnovation is to investigate and offer perspectives on how exnovation can be applied effectively within the intricate and ever‐changing healthcare landscape, particularly in its nursing implications. This exploration aims to delve into the importance of exnovation in healthcare and nursing, emphasising its unique characteristics compared to other industries.

## 2 Background

In the ever‐changing world of organisations and new ideas, exnovation is an important but often ignored concept. However, innovation often results in improvements and new ideas, whereas exnovation is the deliberate removal of old practices, technologies or processes from an organisation (O'Reilly and Tushman 2008). This idea involves a careful and purposeful method of eliminating outdated or unhelpful elements to make room for renewal, efficiency and long‐lasting progress.

Unlike innovation, which gets a lot of attention and resources, exnovation quietly changes how organisations work and helps them adapt to new situations (McGrath 2013). Understanding exnovation is crucial for organisations seeking to streamline their operations, increase agility and maintain relevance in rapidly evolving markets. Exnovation involves deliberate elimination of old processes in healthcare organisations, in terms of innovation, efficiency and sustainable development (Berwick et al. 2008). As health systems must constantly adapt to changing patient needs and technology, (Langley and Denis 2011), recognising and implementing exnovation is essential for keeping high standards of care.

In nursing, exnovation involves identifying and removing outdated practices to improve patient care. Nurses, through ongoing education and advocacy, play a vital role in promoting this process and communication strategies (Iedema et al. 2022). By staying informed about evidence‐based approaches (Melnyk and Fineout‐Overholt 2022), nurses advocate for changes such as revising guidelines and adopting innovative technologies (Elshaug et al. 2017). This proactive approach ensures that nursing practices align with current standards, leading to better outcomes for patients (Ackerman et al. 2020).

This concept analysis aims to examine exnovation in healthcare by uncovering its features, origins, processes, challenges, examples and effects. It seeks to develop strategies to address exnovation and evaluate its impacts on healthcare and society.

## 3 Methodology

The concept of exnovation is scrutinised utilising the Walker and Avant method, employing eight sequential steps. These steps encompass identifying all applications of the concept, determining its defining attributes, pinpointing a model case, recognising instances along the spectrum including borderline, related, contrary, invented and illegitimate cases, identifying antecedents and consequences associated with exnovation and finally, defining empirical referents (Hartley and Knell 2022). Through this systematic approach, a comprehensive examination of exnovation is conducted, facilitating its comprehension and application across various contexts.

### 3.1 Methodology—Application of Walker and Avant's 8‐Step Approach

This study utilised Walker and Avant's (2005) 8‐Step Approach to concept analysis, applying each step as follows:

- Select the concept—‘Exnovation’ was chosen based on its emerging relevance in healthcare and limited exploration in nursing literature.
- Determine the purpose of the analysis—The aim was to clarify the meaning, attributes, antecedents and consequences of exnovation in nursing and healthcare contexts.
- Identify all uses of the concept—A systematic search of PubMed and Scopus was conducted using the keyword ‘exnovation’ combined with relevant MeSH terms (e.g., ‘Healthcare Disinvestment,’ ‘Innovation in Nursing’). No date restrictions were applied. Studies from healthcare, business, administration and related fields were included to capture cross‐sector applications.
- Determine the defining attributes—Literature was read in full and coded for recurring themes and descriptive characteristics of exnovation. Attributes were synthesised through iterative comparison across contexts.
- Identify a model case—A hypothetical healthcare scenario fully embodying the identified attributes was constructed to exemplify exnovation in practice.
- Identify borderline, related, contrary, invented and illegitimate cases—Alternative cases were developed to contrast with the model case, highlighting boundaries and overlaps with related concepts.
- Identify antecedents and consequences—Conditions that precede (antecedents) and result from (consequences) exnovation were extracted directly from the literature and categorised according to their impact on nursing, patient care and organisational processes.
- Define empirical referents—Observable indicators of exnovation in healthcare (e.g., documented removal of outdated practices, policy changes, technology decommissioning) were identified from the literature to facilitate measurement in future research.

This structured application ensured conceptual clarity and allowed for a comprehensive understanding of exnovation in nursing and healthcare.

## 4 Literature Search

When conducting a literature review on ‘exnovation,’ a systematic search approach was employed across PubMed and Scopus. The search strategy was carefully designed to incorporate specific MeSH (medical subject headings) terms in combination with the keyword ‘exnovation’. The chosen MeSH terms comprised ‘Healthcare Disinvestment’, ‘Overtreatment’, ‘Destabilization Of Health Care’, ‘Innovation Bias’, ‘Managerial Innovation’, ‘Innovation in Nursing, “Innovation In Health Care Management”’ and ‘Exnovation’.

The search strategy utilised Boolean operators to broaden the search scope, structured as follows for PubMed: ((healthcare disinvestment) or (overtreatment) or (destabilisation of health care) or (innovation bias) or (managerial innovation) or (innovation in nursing) or (innovation in health care management)) and (exnovation).

Predefined inclusion criteria required that articles be directly relevant to exnovation or conceptually related to its attributes, antecedents, consequences or applications; be published in English; and be peer‐reviewed journal articles, reviews or scholarly book chapters. Grey literature was excluded. No date restrictions were applied to allow examination of the concept's evolution over time. Duplicate records identified across databases were removed before screening.

Following these criteria, all retrieved articles were retained for full‐text analysis. None were excluded due to irrelevance, language or methodological shortcomings, as each contributed unique perspectives or examples pertinent to the concept. Given the exploratory nature of concept analysis, we prioritised breadth and diversity of sources to ensure a comprehensive understanding. This inclusive approach enriched the analysis by incorporating multidisciplinary viewpoints, which strengthened the robustness and applicability of the final conceptual framework.

**FIGURE 1:**
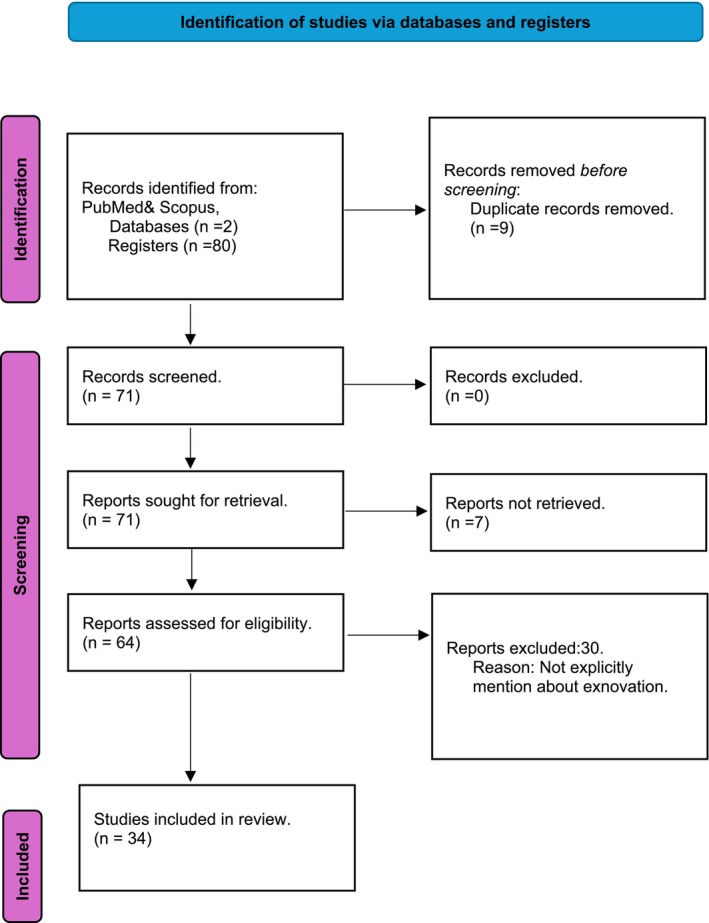
Identification of studies via databases and registers.

The PubMed/Medline search yielded 16 articles: 10 studies focused on innovation and exnovation, while 6 studies specifically addressed exnovation. A parallel search in Scopus yielded 48 studies, with 18 discussing exnovation and 30 covering innovation, some of which explicitly addressed exnovation. All identified articles are selected to develop concepts for writing, including a few specifically on exnovation in healthcare. Additionally, related articles on exnovation in transition, energy, sustainability and administration studies were included. (Figure 1).

## 5 Explanation of the Concept

**FIGURE 2:**
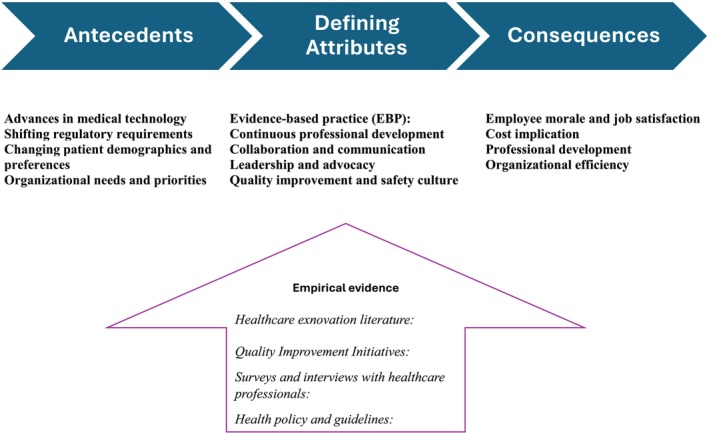
A summary of the key elements of the concept analysis of exnovation.

The term ‘exnovation’ isn't listed in standard dictionaries. To gain a deeper understanding of this concept, we employed two approaches: the onomasiological and semasiological approaches. By combining these approaches, a more comprehensive understanding of what ‘exnovation’ can be formulated (Figure 2).

### 5.1 Phase I—Onomasiological Approach

The onomasiological approach focuses on the process of finding the right word or expression to convey a particular concept or idea about the process of ending up recommending one word over the others (Holbek and Knudsen 2020). In this phase, we utilise this method to discuss the evolution of the term ‘exnovation.’

#### 5.1.1 Evolution of Terminology

The term ‘exnovation’ comes from combining the prefixes ‘ex‐’ meaning ‘out’ or ‘away from’ and ‘innovation,’ meaning a departure from established norms or practices (West and Farr 1990). It was first introduced by John Kimberly in 1981 as ‘the removal of innovation from an organization’ (Kimberly 1981). Exnovation was defined more recently in 1996 as avoiding another innovation particularly when best‐in‐class entities exist. As such, exnovation has found acceptance among different fields such as medicine and management.

#### 5.1.2 From Innovation to Exnovation

Ziegler (2023) describes exnovation as the ‘lesser‐known sibling of innovation’ or its ‘flipside’. One aspect is that exnovating practices and products provide room for new ones, whereas introducing a new product or practice helps eliminate old ones (Davidson 2019). Exnovation refers to the process of phasing out old practices, technologies or processes in different industries to allow new, more efficient methods (Holbek and Knudsen 2020). The spread of ‘innovation’ is more compared to ‘exnovation’ but is now used in different contexts and is more popular.

In recent years, the concept has gained traction in sustainability and transition research, particularly in examining the intentional phase‐out of unsustainable technologies, products and practices. This is especially pertinent in discussions surrounding energy transitions and the cessation of coal usage (Ziegler 2023).

#### 5.1.3 Parallel Definitions of Exnovation

##### 5.1.3.1 Content Definition

An exnovation is any idea, practice or material artefact in the adoption unit that needs to be removed or modified to make room for innovation (Zaltman et al. 1973).

##### 5.1.3.2 Process Definition

An exnovation process is a sequence of linked events, actions and activities undertaken to remove or modify ideas, practices or material artefacts to make room for innovation (Bessant 2005).

##### 5.1.3.3 Phenomenological Definition

Exnovation means that something is being removed or modified to allow space for innovation (Holbek and Knudsen 2020).

### 5.2 Phase 2‐ Semasiological Approach

The semasiological approach underscores how a single concept, such as exnovation in our case, can lead to diverse interpretations, emphasising the necessity of pursuing a cohesive comprehension of the term (Holbek and Knudsen 2020). Through this approach, we discussed the applicability of the concept in various settings.

Exnovation has been identified in multiple sectors, with notable advances in the two main clusters of adaptation/strength/sustainability studies and health care but limited interactions between these clusters (Verger et al. 2024). Moreover, these two clusters use the term ‘exnovation’ in different contexts.

#### 5.2.1 First Cluster

In the realm of information technology (IT), exnovation focuses on changing technical standards, increasing system performance by dropping outdated software and unsupported assemblies, and reducing security risks. (Bhattacherjee et al. 2018). Similarly, in data management, exnovation means that maintaining data integrity may involve saving or deleting outdated data structures, databases or files, optimising storage space and facilitating data retrieval (Ravichandran et al. 2017).

In manufacturing and production, emphasis has been placed on the principle of exnovation. This approach prioritises a focus on reducing waste, creating customer value and seeking continuous improvement in processes (Bate and Robert 2023). Moreover, facilities can actively engage in exnovation using modern technology; it will replace old machinery or processes (Womack et al. 2007). Such improvements increase efficiency, improve energy efficiency and improve product quality. By integrating exnovation practices within lean manufacturing frameworks, organisations can improve operational efficiency and remain competitive in the face of evolving market trends (Adler‐Milstein et al. 2019).

In realm of business and organisational management, businesses can exnovating by reviewing and modifying internal policies, procedures or procedures to adopt evolving marketing condition, customer requirements or regulatory requirements (Davenport and Harris 2007). When it comes to manufacturing exnovation involves the elimination of inefficient products or materials to better allocate resources to innovate with greater market potential or system compatibility (Cooper 2008).

Within the education sector, exnovation in education may take the form of course materials, textbooks or other instructional methods to reflect current knowledge, best practices and educational standards (Desimone et al. 2013). Old educational technologies or digital learning tools can be phased out and replaced with new interactive, engaging, adaptive methods that better support student learning outcomes (Kinshuk et al. 2016).

In environmental conservation, alternatives created in environmental protection may include reducing or eliminating the use of single‐use plastics or harmful eco‐friendly materials in favour of environmentally unacceptable (Bazaz 2023). Furthermore, exnovation can include reevaluating resource‐intensive practices or technologies in industries like agriculture or energy production to minimise environmental impact and promote sustainability (Bocken et al. 2013).

Exnovation can also occur in cultural contexts, where social norms, beliefs or practices change over time. Some cultural traditions, rituals or customs may be gradually shed or modified as societies evolve and adapt to new circumstances (Boyd and Richerson 2005). For example, the decline of traditional manual processes in favour of automated production methods represents a new development in cultural practices.

#### 5.2.2 Second Cluster

Exnovation, and the phasing out of old treatments or methods through new, more effective methods supported by evidence‐based medicine (Greenhalgh et al. 2017). These initiatives extend to health systems, where exnovation can be used to streamline workflows, eliminate redundant paperwork or supplement existing electronic health record (EHR) systems to improve efficiency and reduce health costs (World Health Organization 2010). Therefore, those with better diagnostic accuracy and patient comfort manage costs (Adler‐Milstein et al. 2019) and aid in chronic care management over time (Rodriguez et al. 2016).

Similarly, exnovation in health research is characterised by a practice‐based definition. Mesman (2017) describes exnovation as enhancing existing practices by examining the underlying mechanisms that are already effective (Mesman 2017). Therefore, exnovation, along with advancements in medical technology, collectively contributes to improving the quality and efficiency of health care (Rodriguez et al. 2016).

## 6 Attributes

An attribute is a characteristic or quality inherent to a concept, observed consistently across various contexts where the concept is discussed (Walker and Avant 2005). While specific characteristics of ‘exnovation’ are not exclusively described, the concept is often discussed in the broader literature on innovation management, technology adoption and on organisational change. Here are some key references that discuss related concepts, contributing to understanding the defining attributes of exnovation:

- *Deliberate elimination or reduction*: Exnovation involves a conscious decision to eliminate or reduce old practices, technologies, products or services (Christensen 1997).
- *Substitution or abandonment*: Involves replacing existing processes or systems with more effective ones or abandoning them altogether (Henderson and Clark 1990).
- *Adaptive flexibility*: Exnovation occurs in response to changes in circumstances, such as technological advances, changes in market demand, or evolving of social norms (Tushman and O'Reilly 1996).
- *Improvement or change*: The goal of exnovation is to improve efficiency, sustainability or relevance by eliminating outdated and inefficient processes.
- *Strategic decision‐making*: Exnovation involves planning and decision‐making processes to align organisational or societal behaviour with current needs, goals or values (Christensen 1997; Tushman and O'Reilly 1996).
- *Ongoing process*: Exnovation is an ongoing process that may occur repeatedly over time as circumstances change and new opportunities or challenges emerge (Henderson and Clark 1990).

Moreover, various methodologies are employed to convey a similar concept of exnovation (Table 1). Understanding the disparities among these methodologies can deepen one's comprehension of the exnovation concept.

**TABLE 1:** Various concepts resembling exnovation.

Concept	Definition	Key principles/Approach
Lean	Philosophy focused on maximising customer value while minimising waste (Cohen 2018)	Emphasising continuous improvement, respect for people and elimination of non‐value‐added activities for efficient processes
Six sigma	Data‐driven method for reducing defects and variations in products, services and processes (Yang et al. 2003)	Using a structured approach (DMAIC) to identify and eliminate errors, aiming for high quality and performance levels
Kaizen	Kaizen is a Japanese term for continuous improvement. It involves making small, incremental changes to processes, systems and workflows to achieve ongoing improvements in quality, efficiency and effectiveness (Macduff 2020)	Kaizen emphasises the importance of identifying and addressing inefficiencies or shortcomings to drive positive change over time
Agile methodology	Agile Methodology is a flexible approach to developing software and managing projects. It focuses on collaboration and adapting to changes quickly. It breaks projects into small tasks, regularly reviews progress and adjusts plans based on feedback and new needs (Beckett et al. 2021)	Agile shares similarities with exnovation in its focus on adapting to changing circumstances and continuously improving processes to achieve better outcomes

This session also examines the qualities of exnovation within healthcare, especially in nursing, encapsulating various essential attributes.

- *Evidence‐based practice* (*EBP*): Exnovation in nursing involves adherence to evidence‐based guidelines and the integration of the latest research findings into clinical practice. Nurses critically evaluate existing practices, aiming to replace outdated or ineffective medications with interventions supported by current evidence (Melnyk and Fineout‐Overholt 2022).
- *Continuous professional development*: Nurses engaged in exnovation are dedicated to continuous learning and professional development. They actively seek new knowledge, skills and innovations in nursing practice, staying updated on advances in health care delivery to provide optimal patient care (Bate and Robert 2023).
- *Patient‐centred care*: Exnovation in nursing prioritises personalised care to meet each patient's unique needs, preferences and values. Nurses focus on optimising practices to align with patient‐centred principles to promote overall well‐being and enhance patient outcomes (Hartley and Knell 2022).
- *Collaboration and communication*: Exnovation involves nurses collaborating with interdisciplinary healthcare teams to facilitate the exchange of ideas and best practices (Dieckmann et al. 2017). Effective communication and teamwork are essential to identifying outdated practices, implementing change and fostering a culture of continuous improvement (Iedema et al. 2022).
- *Quality improvement and safety culture*: Exnovation is embedded in a culture of quality improvement and patient safety. Nurses systematically participate in maintaining and improving care quality, prioritising patient safety and reducing risks associated with outdated practices (Berwick and Shojania 2015).
- *Leadership and advocacy*: Nurses demonstrate leadership qualities by advocating for the elimination of outdated practices and supporting the adoption of new nursing care approaches. They play a critical role in driving change, influencing policy decisions and promoting excellence in nursing practice (Harrington 2015).

## 7 Model Case

A model case serves as a practical and comprehensive example that embodies all the features of a given concept (Brush et al. 2011). It is an epitome that can be visualised or experienced like any tangible object. This article outlines how an example of exnovation could theoretically exist by providing an instance using it. Subsequently, we will present a model case that encapsulates all the defining qualities of exnovation.

### 7.1 Scenario

A large hospital decides to shift its nursing staff to 12‐h shifts to reduce costs, increase flexibility and improve employee satisfaction. At first, the innovation seemed promising, with nurses forever hoping to have more consecutive days off and management predicting the potential economic benefits of reduced commutes. These changes have been shown to increase job satisfaction, reduce turnover and, finally, make patients well cared for.

However, several issues relating to order, life and well‐being emerged after the implementation of the 12‐h shift. Nurses reported feeling tired and burnt out due to long working hours, which, despite decreased alertness and impaired patient safety, strained social and family relationships during a period in which nurses struggled to balance work and personal commitments. In addition, irregular schedules made it difficult for nurses to maintain work‐life balance, leading to increased stress and decreased overall well‐being.

Because of these challenges, hospital management recognised the need for changes to the 12‐h work schedule. Despite the initial intention to improve economic efficiency and staff satisfaction, the negative effects on nurse well‐being and patient care outweighed the perceived benefits. As a result, the hospital returned to a conventional business model, prioritising the needs of staff and patients over cost‐cutting measures.

## 8 Additional Cases

In a concept analysis, exploring cases that are highly contrasting or closely resembling the concept of interest helps refine the defining attributes (Brush et al. 2011; Walker and Avant 2005). Below, we discuss the borderline, related and contradictory cases, which help to understand the applicability in nursing.

### 8.1 Borderline Case

#### 8.1.1 Scenario

In a rural nursing home, there's a consideration to discontinue the primary caregiver model due to staffing shortages and evolving patient needs. While this model offers benefits like consistent and personalised care, it's challenging to sustain.

On the one side, the primary caregiver model has proven effective in providing consistent and personalised care. Assigning residents to specific care providers builds trust and knowledge, positively impacting residents' well‐being. Additionally, it promotes communication and collaboration among caregivers, improving care management.

However, challenges arise during the high‐demand period or staff turnover. Relying on a small number of specialists can strain funding, and staff shortages can affect care quality and consistency. Considering these operational issues, the nursing home must weigh the benefits against the risks of discontinuing the primary caregiver model. Maintaining continuity of care and the resident‐staff relationship is crucial, but adapting to staffing challenges is necessary for quality care.

The decision to change the caregivers' hiring methods requires careful consideration of the pros and cons. The nursing home should evaluate how changes may impact resident care, staff satisfaction and overall efficiency. Input from residents, families and staff is essential to address concerns and ensure a smooth transition. The goal is to adapt to new challenges while retaining the strengths of the current caregiver system and providing excellent care.

### 8.2 Related Case

#### 8.2.1 Scenario

A nursing school is incorporating exnovation into its new curriculum design to better prepare students for modern health care. This strategy seeks to remove outdated materials and teaching methods from nursing curricula while integrating EBPs, fostering interprofessional collaboration and providing simulation‐based learning experiences. By aligning the curriculum with current healthcare information and skills, nursing schools enhance student readiness for the growing nursing profession.

This case highlights how exnovation in nursing can be linked to educational innovation efforts aimed at increasing the quality and relevance of nursing education. By eliminating outdated materials and teaching methods and integrating modern teaching strategies, schools of nursing equip students with the knowledge and skills needed to succeed in modern health care.

### 8.3 Contradictory Case

#### 8.3.1 Scenario

A district hospital nursing team chose to carry out its new policy of monitoring and enforcing compulsory hand hygiene among nursing staff despite the abundant evidence of the role, especially the role of hand hygiene in preventing health‐associated infection. They justify this decision by arguing that constant monitoring creates a sense of distrust among nurses and infringes upon their autonomy.

By skipping routine hand hygiene audits and relying solely on staff self‐reporting, a nursing team essentially eliminates the accountability system that ensures compliance with hand hygiene policies. While the leadership perceives this as a measure to empower nurses and promote autonomy, it neglects the critical aspect of patient safety and infection prevention.

This decision represents a paradox between EBP and organisational culture concepts. While evidence supports the importance of adherence to hand hygiene in infection prevention, leadership emphasises perceived cultural disregard for established infection control measures. This compromises patient safety by increasing the risk of healthcare‐associated infections among the nursing team, prioritising perceived autonomy over patient well‐being.

### 8.4 Analysis

This contrasting case underscores the risks associated with deviating from EBPs in nursing, driven by concerns about perceived organisational culture. By overlooking established infection control protocols and failing to monitor hand hygiene compliance, the nursing unit heightens the risk of healthcare‐associated infections and jeopardises patient safety.

## 9 Antecedents

Antecedents are the conditions or factors that precede or contribute to the concept of exnovation (Walker and Avant 2005). In medical and nursing contexts, several key antecedents of exnovation have been identified in the literature:

- Advances in medical technology: Rapid advancements in medical technology often render old methods obsolete, prompting healthcare organisations to adopt new technologies that better serve patients (Kim and Park 2019).
- Shifting regulatory requirements: Changes in healthcare regulations, standards or accreditation requirements are often indicators of exnovation. The revised guidelines may require modifications to existing practices to ensure compliance and motivate organisations to reassess and update their policies accordingly (Huang et al. 2020).
- Emerging EBPs: Continued advances in medical knowledge and EBPs are driving exnovation efforts in health care (Kaplan et al. 2018). New research findings and clinical evidence often prompt organisations to rethink established practices and incorporate new guidelines into their operations (Braithwaite et al. 2017).
- Changing patient demographics and preferences: Changing patient demographics, preferences and expectations can also trigger exnovation initiatives. Healthcare organisations may need to adapt their practices to meet the needs of different patients or to meet the demands of patients for new methods of care delivery (Say et al. 2006).
- Organisational needs and priorities: Internal factors such as financial constraints, labour shortages or strategic priorities play an important role in determining exnovation priorities (Egbu 2005). Organisations may initiate exnovation efforts to improve productivity, increase employee satisfaction or address resource constraints (Barr and Nathenson 2022).

## 10 Consequences

Consequences are the events that happen after the concept has occurred (Walker and Avant 2005). The consequences of exnovation in medical and nursing fields can have far‐reaching effects on patient care, organisational efficiency and professional development.

- Quality of patient care: Exnovation can positively and negatively affect the quality of patient care. It may lead to the adoption of more effective influence practices and improve patient outcomes (Fisher et al. 2016). However, if not properly managed, exnovation can lead to the elimination of useful practices or technologies, potentially compromising patient safety and quality of care (Leggat et al. 2019).
- Organisational efficiency: Exnovation can streamline processes and reduce unnecessary tasks, leading to increased productivity in healthcare organisations (Adler‐Milstein et al. 2019). Eliminating outdated or irrelevant practices can reallocate resources more efficiently (Cooper 2008), and employee time and financial resources can be used more efficiently (Ariza‐Montes et al. 2016).
- Professional development: Exnovation often involves changes in professional practices and skill sets among healthcare professionals. Nurses and other healthcare professionals may need to undergo training or re‐education to adapt to new systems or technologies because of exnovation (Porter et al. 2016). While this can lead to professional growth and development, it can also pose challenges in terms of time and resource allocation for training (Van Bogaert et al. 2017).
- Employee morale and job satisfaction: Exnovation can impact employee morale and job satisfaction (Willems 2021). Effective exnovation can reduce administrative burdens and enable healthcare providers to focus more on direct patient care, potentially increasing job satisfaction (Aiken et al. 2012). However, if perceived as disruptive or poorly managed, restructuring efforts can lead to frustration and dissatisfaction among employees (Ariza‐Montes et al. 2016).
- Cost implication: Exnovation carries financial implications for healthcare organisations. While some exnovation efforts may result in cost savings by eliminating unnecessary practices or technologies, others may require investments in new equipment, training programmes or implementation technologies (Porter et al. 2016). Healthcare organisations must carefully weigh the potential cost savings against exnovation (Willems 2021) and the long‐term benefits of the initial investment (Champagne 2002).

## 11 Empirical Referents

Empirical referents are events that serve as evidence that the concept has occurred (Walker and Avant 2005). While research on exnovation in medicine and nursing science may be relatively limited compared to fields like technology or business, there are still avenues to explore for empirical references:

- *Healthcare exnovation literature*:
  Although the term ‘exnovation’ is not widely used in the journals, it often refers to the adoption, dissemination and phasing out of innovations in healthcare settings (Kaplan et al. 2012).
- *Case studies of healthcare organisations*:
  Studies or research papers that explore how healthcare organisations have utilised outdated technologies or practices, especially as they phase them out (Gawande 2010). Examples might include the adoption of EHRs, the integration of telemedicine or the use of EBPs in clinical care.
- *Quality Improvement Initiatives*:
  Quality improvement efforts frequently involve revising existing processes and systems to enhance patient outcomes and operational efficiency (Christensen et al. 2009). Six Sigma and Lean are indeed quality improvement initiatives commonly used in various industries, including healthcare.
- *Surveys and interviews with healthcare professionals*:
  Qualitative surveys, interviews or discussions with healthcare providers, practitioners and policymakers can provide empirical data about the factors influencing the decision to adopt medical technology and treatments or accept, continue or abandon a particular role (Rogers 2003).
- *Health policy and guidelines*:
  Government agencies, professional organisations and healthcare systems often issue guidelines and policies that impact the adoption and abandonment of medical or technology products (World Health Organization 2019). Research focusing on changes in healthcare policy or guideline adherence may offer insights into instances of exnovation within clinical settings.
- *Medical device and pharmaceutical industries*:
  Studies examining the life cycle of medical devices or pharmaceutical products may include cases of obsolescence or withdrawal due to safety concerns, technological advances or changes in clinical practice standards (Ertz and Patrick 2020).

While empirical references specifically focusing on exnovation in medicine and nursing science may be limited, examining these pathways can provide valuable insights into the innovation and change that occur in health care settings.

## 12 Discussion

This analysis delves into the concept of ‘exnovation’, exploring its attributes, antecedents and consequences. Through a comprehensive analysis, the study seeks to identify the applicability of the exnovation in various settings, particularly within the nursing and healthcare sector. Its primary objective is to develop practical and relevant definitions of performance that can be used in nursing or medicine.

An important aspect of the analysis is to determine the dynamics and evolution of exnovation. It emphasises how exnovation is different from innovation. To accurately define the concept of ‘exnovation,’ it is necessary to understand how it differs from innovation.

Understanding the distinct characteristics of exnovation in comparison to innovation is crucial for comprehending its role in organisational change. It underscores the significance of distinguishing between exnovation and innovation, highlighting their individual traits and objectives. As both terms are commonly used in the realm of innovative endeavours, it's essential to recognise that defining exnovation without reference to innovation is incomplete. Table 2 provides a detailed comparison of the differences between exnovation and innovation.

**TABLE 2:** Comparison of the differences between exnovation and innovation.

Aspect	Innovation	Exnovation
Definition	The introduction of new concepts, methods, procedures or processes that create value or bring about positive chang	The intentional reversal or discontinuation of improvements that have been formerly applied due to unsatisfactory outcomes or unexpected negative consequences
Process	Involves the improvement and implementation of novel answers to address existing demanding situations or possibilities	Occurs while an innovation fails to provide the preferred outcomes or leads to unforeseen poor results, prompting a decision to reverse or discontinue it
Goal	Drives progress, growth and improvement by introducing something new or distinctive that enhances efficiency, effectiveness or competitiveness	Addresses issues arising from unsuccessful or unsustainable innovations by reverting to previous practices or adopting alternative approaches
Outcome	Introduces new ideas or solutions to drive positive change and enhance organisational performance	Rectifies or replaces unsuccessful innovations to ensure that they do not negatively impact organisational effectiveness or hinder progress

Moreover, the study acknowledges that the experience of exnovation may vary across contexts and locations, making it important to understand this concept in the context of technology and healthcare applications, especially in nursing.

### 12.1 Comprehensive Operational Definition of Exnovation in Health Care

*The comprehensive operational definition of exnovation refers to the deliberate process focusing on identifying, evaluating and abandoning outdated or ineffective practices, procedures, technologies or systems within health care*.

### 12.2 Comprehensive Operational Definition of Exnovation in Nursing

*Exnovation in nursing is a systematic approach to identifying and eliminating the elements of nursing practice that do not contribute to desired outcomes or are inconsistent with current standards of care. The antecedents to exnovation include robust advanced technology and empowered patients, while the consequences elevate professional development and satisfaction*.

Based on the definition, exnovation has three main elements in action:

*Identification of Ineffectiveness*:

Exnovation involves recognising current practices that are ineffective, inefficient or outdated and need a change.

*Deliberate Abandonment*:

It entails a purposeful process of eliminating or gradually phasing out outdated practices, technologies or methods.

*Adoption of Superior Alternatives*:

Exnovation aims to replace obsolete practices with newer, more efficient alternatives that offer improved effectiveness and outcomes.

Moreover, exnovation in nursing includes identifying the characteristics, antecedents and consequences associated with obsolete practices and implementing strategies to facilitate their elimination or transfer. This operational definition emphasises the dynamic and evolving nature of exnovation, acknowledging the need for ongoing evaluation and adaptation to optimise healthcare delivery.

## 13 Future Implication

A clear understanding of exnovation could transform nursing practice, education and policy, leading to a more empowered and proficient nursing workforce and improved patient outcomes. The thorough comprehension and definition of ‘exnovation’ could bring about important changes across different areas in the future.

### 13.1 Efficiency and Streamlining

Exnovation in nursing involves identifying and eliminating outdated or inefficient processes, thereby streamlining work and promoting health system delivery.

### 13.2 Resource Optimization

Exnovation can help in improving the quality of resources by eliminating redundant or unnecessary practices and maintaining resources for better allocation.

### 13.3 Focus on Patient‐Centred Care

Exnovation can enable nurses by reducing time on outdated administrative tasks or processes; nurses can focus more on patient‐centred care, removing barriers to quality care, allowing nurses to devote more time and attention to patients' individual needs and preferences.

### 13.4 Adaptation to Technological Advances

Exnovation can involve phasing out outdated technologies or processes that prevent the integration of new, more advanced technologies. This allows nursing facilities to keep abreast of technological advances, increasing the quality and safety of patient care.

### 13.5 Promotion of Innovation Culture

Embracing exnovation in nursing can foster a culture of innovation and continuous improvement. By encouraging nurses to critically assess existing practices and identify areas for elimination or improvement, healthcare organisations can foster a culture that fosters innovation and better adapt to the changing health landscape.

### 13.6 Risk Management and Patient Safety

Exnovation initiatives aimed at eliminating such practices can enhance patient safety and reduce the likelihood of adverse events, thereby improving overall health care.

### 13.7 Professional Development

Exnovation requires nurses to engage in critically reflecting on and evaluating their practice, contributing to continuous professional development. It encourages nurses to stay abreast of current best practices, research findings and technological developments in their field.

## 14 Limitation

While this study offers valuable insights into the concept of exnovation, it also has limitations that need to be considered.

Firstly, there may be a scarcity of empirical studies specifically focusing on exnovation in nursing and healthcare. This lack of research can hinder a more profound understanding and exploration of the concept.

Secondly, the understanding of exnovation may vary depending on the specific context of nursing and healthcare practices. What is new in one context may be irrelevant or redundant in another. Moreover, exnovation intersects with various disciplines beyond nursing and healthcare, such as organisational management and technology. This interdisciplinary nature adds challenges to its evaluation and application in health care contexts.

It's important to note that concept analysis relies heavily on interpretation, which can introduce subjectivity. Different researchers may have varying interpretations of the concept of exnovation, further complicating its understanding and application in practice.

## 15 Conclusion

In conclusion, this analysis sheds light on what exnovation is and how the practice might act in various contexts. A comprehensive understanding of this concept underscores its significance in identifying and eliminating outdated practices to improve patient care. This, in turn, contributes to optimising practice redesign, education and policy development, all geared towards enhancing the effectiveness of the nursing workforce. Furthermore, as a relatively new concept in nursing and medical practice, further research is necessary to address the identified limitations and formulate practical guidelines for the successful integrating of exnovation into nursing and healthcare environments.

## Author Contributions

A.J.N. and G.V.J.: Made substantial contributions to conception and design, or acquisition of data, or analysis and interpretation of data. A.J.N., G.V.J., K.M., J.K., F.A., A.M.A.A. and A.A.A.: Involved in drafting the manuscript or revising it critically for important intellectual content. A.J.N., G.V.J., K.M., J.K., F.A., A.M.A.A. and A.A.A.: Given final approval of the version to be published. Each author should have participated sufficiently in the work to take public responsibility for appropriate portions of the content. A.J.N. and G.V.J.: Agreed to be accountable for all aspects of the work in ensuring that questions related to the accuracy or integrity of any part of the work are appropriately investigated and resolved.

## Ethics Statement

The authors have nothing to report.

## Conflicts of Interest

The authors declare no conflicts of interest.

## Data Availability

Data sharing not applicable to this article as no datasets were generated or analysed during the current study.
